# Melatonin inhibits osteoclastogenesis via RANKL/OPG suppression mediated by Rev‐Erbα in osteoblasts

**DOI:** 10.1111/jcmm.17440

**Published:** 2022-06-21

**Authors:** Yihao Tian, Jian Ming

**Affiliations:** ^1^ Department of Pathology General Hospital of Northern Theater Command Shenyang China

**Keywords:** co‐culture, diabetic osteoporosis, melatonin, mir‐882, osteoclastogenesis, rev‐Erbα

## Abstract

Diabetic osteoporosis is secondary osteoporosis and a serious complication of diabetes with a high incidence rate and poor prognosis. The specific mechanism of diabetic osteoporosis is unclear, and prevention and treatment options are limited. Recently, melatonin has been found to prevent and treat diabetic osteoporosis. Herein, we investigated the mechanism whereby melatonin inhibits osteoclastogenesis and identified a new target for osteoporosis treatment. We established an in vitro osteoblast–osteoclast co‐culture system as a diabetic osteoporosis model. Osteoclastogenesis was determined using tartrate‐resistant acid phosphatase staining and cathepsin K expression. Real‐time PCR was used to ascertain expression of microRNA mir‐882, targeting Rev‐Erbα. Western blotting was performed to detect the expression of Rev‐Erbα, receptor activator of NF‐kB ligand (RANKL), and osteoprotegerin (OPG), and ELISA was utilized to analyse the secreted form of RANKL. High glucose promoted osteoclastogenesis and elevated the RANKL/OPG ratio in osteoblasts, while melatonin reversed these effects. High glucose inhibited Rev‐Erbα expression, while melatonin promoted its expression. Conversely, high glucose promoted mir‐882 expression, while melatonin inhibited it. We infer that melatonin inhibits RANKL expression in osteoblasts via the mir‐882/Rev‐Erbα axis, thus inhibiting osteoclastogenesis. Our findings provide insights into diabetic osteoporosis and identify a new therapeutic target for osteoporosis.

## 1 INTRODUCTION

Concurrent with improvements in living standards and changes in living habits, the global incidence of diabetes has been increasing yearly.^1^ Several diabetes‐related complications, including diabetic osteoporosis, pose severe threats to the well‐being of people with diabetes.^2^, ^3^ Although the pathogenesis of diabetic osteoporosis is unclear, recent studies have suggested it may be related to the influence of a high‐glucose environment on metabolism, subsequently affecting bone formation, and may be realized through gene polymorphism changes.^4^ Diabetic osteoporosis may also be caused by excessive reactive oxygen species (ROS) production^5^, ^6^, ^7^ and advanced glycation end products.^5^, ^8^, ^9^, ^10^ In addition, hyperglycaemia can promote the nonenzymatic glycosylation of diverse bone matrix proteins, such as type I collagen, resulting in bone damage.^11^ Moreover, insulin promotes osteoblast growth, as the osteoblast surface contains insulin receptors; however, insulin levels decrease in diabetic patients, affecting osteoblast growth.^12^, ^13^, ^14^ Diabetes may also cause calcium loss, with low blood calcium levels leading to secondary hyperparathyroidism, activating osteoclasts.^15^ Furthermore, diabetes can lead to peripheral blood vessel disease, neuropathy, and kidney disease, among other disorders, as precursors of osteoporosis.^16^ Kidney damage caused by diabetes can result in decreased hydroxylase activity, leading to reduced vitamin D levels.^15^ There are currently no specific drugs for the treatment of diabetic osteoporosis; however, recent studies have demonstrated potency of melatonin in hampering and treating diabetic osteoporosis.^17^, ^18^

Melatonin has been more consistently shown to be anabolic in multiple studies using mouse genetic models and also physiological doses of melatonin.^19^, ^20^ Melatonin is a rhythmically secreted hormone important for maintaining normal circadian rhythm in organisms.^21^ In organisms, several clock genes encode clock or rhythm proteins,^22^ including Clock/Bmal complex, Per/Cry complex, Rev‐Erb and Ror. Clock/Bmal complex and Ror are positive circadian clock regulators, while Per/Cry complex and Rev‐Erb are negative regulators. The positive and negative regulatory factors form a feedback loop and regulate each other to maintain normal biological clock rhythm.^23^, ^24^ Rev‐Erb is also a nuclear transcription inhibitor that exerts biological effects by inhibiting transcription.^25^ Rev‐Erb, in both its α and β forms, is considered an orphan receptor as no endogenous ligand in vivo has been identified. Rev‐Erb is expressed in adipose tissues, bones and muscles,^26^, ^27^, ^28^ and several studies have confirmed that it has diverse functions. For example, Rev‐Erb is involved in cell metabolism, gene transcription and other physiological processes,^29^, ^30^ as well as pathological processes, such as renal carcinoma,^31^ glioblastoma multiforme,^32^ autoimmune diseases^33^ and hepatitis C.^34^ The effect of melatonin on bone mass varies under diabetic osteoporosis conditions. Exogenous melatonin can prevent type 1 diabetes mellitus‐induced bone loss by inhibiting senescence.^35^ Melatonin can also suppress ferroptosis induced by high glucose via activation of the Nrf2/HO‐1 signalling pathway in type 2 diabetic osteoporosis.^36^

Healthy bone in organisms depends on a dynamic equilibrium between bone production by osteoblasts and bone absorption by osteoclasts. Disturbance of this balance results in bone diseases, with increased bone formation leading to osteosclerosis and increased bone resorption leading to osteoporosis.^37^, ^38^ Osteoblasts and osteoclasts regulate and interact with each other through both direct contact and paracrine signalling.^39^ Receptor activator of NF‐κB ligand (RANKL), osteoprotegerin (OPG) and other factors secreted by osteoblasts act on osteoclasts and regulate their proliferation and differentiation.^40^, ^41^, ^42^ RANK expressed on the surface of osteoclasts receives information from osteoblasts by binding to RANKL. At the same time, osteoclasts can act on osteoblasts through ATPase H+ Transporting V0 Subunit D2 (ATP6V0D2), complement component 3a, signal transducer 4D, or microRNA.^43^, ^44^ Therefore, establishing an in vitro osteoblast–osteoclast co‐culture system as a model to simulate the in vivo environment is useful to study the mechanisms related to osteoporosis and other bone diseases. This approach can provide results that are easily translated to clinical settings. In the current study, we assessed the biological function of Rev‐Erbα in bone tissue with respect to inhibition of osteoclastogenesis and explored the specific underlying mechanisms.

## 2 MATERIALS AND METHODS

### 2.1 Cell culture

The osteoblastic MC3T3‐E1 and macrophage‐like RAW264.7 murine cell lines were obtained from The Cell Bank of Type Culture Collection of the Chinese Academy of Sciences. The MC3T3‐E1 cells were cultured in α‐MEM medium containing 10% serum and 1% penicillin/streptomycin (HyClone, GE Healthcare). The RAW264.7 cells were cultured in DMEM containing 10% serum and 1% penicillin/streptomycin (HyClone, GE Healthcare). All cells were incubated at 37°C in an incubator with 95% air, 5% carbon dioxide and maximum humidity. Culture media were changed every day, and the cells were passaged every 2 days. MC3T3‐E1 cells were trypsinized before passage, and RAW264.7 cells were aspirated and transferred using a pipette without trypsin digestion. The cells were starved for 24 h in serum‐free media before the experiments were performed.

### 2.2 Osteoblast/osteoclast co‐culture

The co‐culture system was established using a Transwell chamber separated by a 0.4 μm polyethylene terephthalate (PET) membrane. MC3T3‐E1 and RAW264.7 cells were placed in the lower and upper layers of the chamber, respectively, at a 5:1 ratio. The cells were cultured in α‐MEM containing 10% serum and 1% penicillin/streptomycin at 37 °C with 95% air, 5% carbon dioxide and maximum humidity. Then, 30 ng/mL RANKL (R&D Systems, Inc. USA) and 10 ng/mL macrophage colony‐stimulating factor (M‐CSF; R&D Systems, Inc.) were added to initiate osteoclast differentiation.

First, the use of high glucose alone without the assistance of RANKL and M‐CSF could not produce sufficient concentration of RANKL to induce osteoclast differentiation. Second, RANKL measured by Enzyme‐linked immunosorbent assay (ELISA) is not always released by osteoblasts, and some of them are exogenous. However, the same concentration of exogenous RANKL was added in each group in advance, so that the final observation index is the trend of differences between groups.

Osteoblasts were transfected first. After 6–8 h, the fluid was changed and then co‐cultured with osteoclasts. There may be any direct effect of the above axis on osteoclast/precursors. This study mainly focuses on the changes in RANKL/OPG, and it is known, that RANKL/OPG promotes osteoclastogenesis. Although osteoclastogenesis may be directly affected by melatonin, this study detected the most important inducing factor in the early stage of osteoclastogenesis, RANKL/OPG. Therefore, even if it directly affects osteoclast/precursors, the result of osteoclastogenesis is the superposition of RANKL/OPG and direct effect.

### 2.3 RNA oligonucleotide synthesis and transfection

MicroRNA (miRNA) mir‐882 mimics, mir‐882 negative control (NC), mir‐882 inhibitor and mir‐882 inhibitor NC were purchased from Shanghai GenePharma Pharmaceutical Technology Co., Ltd. MC3T3‐E1 cells were transfected with the mimics, inhibitors or NCs using Lipofectamine 2000 (Invitrogen; Thermo Fisher Scientific, Inc.), and successfully transfected cells were then co‐cultured with RAW264.7 cells.

### 2.4 Cell counting kit (CCK)‐8 assay

MC3T3‐E1 cells were seeded into 96‐well plates (5 × 10^3^ cells/well). After incubating for 24 h to allow cell adhesion, 1, 10, and 100 μM melatonin (Sigma‐Aldrich, Merck KGaA), 5, 10, and 15 μM sr9009 (MedChemExpress, Monmouth Junction), or 5, 10, and 15 μM sr8278 (MedChemExpress) were added to the cells. Cellular activity was assessed 48 h post‐treatment using the CCK‐8 assay (Dojindo Molecular Technologies, Inc.) according to the manufacturer's instructions. Formazan was measured at 450 nm using an ELx808 microplate reader (BioTek Instruments, Inc.; Agilent Technologies). Cell viability was calculated as an optical density (OD) value.

### 2.5 Tartrate‐resistant acid phosphatase (TRAP) staining

RAW264.7 cells in the upper Transwell chamber were washed several times with phosphate‐buffered saline (PBS) after discarding the spent culture medium and then fixed with paraformaldehyde at 37°C for 15 min. TRAP staining was then performed according to the manufacturer's instructions (Sigma‐Aldrich; Merck KGaA; cat. no. 387). Briefly, TRAP working solution was added after the fixative was removed and the cells were incubated for 60 min in a water bath at 37°C. The staining solution was then discarded, and the cells were washed twice with PBS. Thereafter, the cell nuclei were stained with haematoxylin for 10 min, and cells were washed twice with PBS. Cell images were acquired using a fluorescence electron microscope (Nikon Corporation; magnification, 200× and 400×). Cells containing three or more nuclei were considered TRAP‐positive osteoclasts.

### 2.6 Western blotting

Treated cells were placed on ice and lysed with RIPA buffer containing 1% phenylmethanesulfonyl fluoride (PMSF; Beyotime Institute of Biotechnology, China) for 30 min. The lysed samples were centrifuged at 12,000 × *g* for 30 min at 4°C. A bicinchoninic acid (BCA) kit (Beyotime Institute of Biotechnology) was used to quantify the protein and 3 μg/μL of protein was loaded onto a 10% resolving gel and separated by sodium dodecyl sulphate–polyacrylamide gel electrophoresis (SDS‐PAGE) at a constant voltage of 80 V. The separated proteins were transferred to polyvinylidene fluoride (PVDF) membrane at a constant current of 200 mA for 1 h. The membrane was blocked with 5% bovine serum albumin (BSA) for 2 h at 25°C and then incubated with the relevant primary antibodies at 4°C overnight. The following primary antibodies were used: Anti‐cathepsin K (cat. no. ab19027, 1:1000; Abcam), anti‐nuclear receptor subfamily 1 group D member 1 (NR1D1/Rev‐Erbα; cat. no. ab174309, 1:5000; Abcam), anti‐RANKL (cat. no. ab45039, 1:500; Abcam), anti‐OPG (cat. no. ab203061, 1:300; Abcam), and anti‐GAPDH (cat. no. 10494‐1‐AP, 1:10,000; ProteinTech Group, Inc.). The membrane was washed three times using tris‐buffered saline with 0.1% Tween 20 (TBST) and incubated with the secondary antibody (1:10,000; ProteinTech Group, Inc.) at 25 °C for 2 h. The membrane was washed three times with TBST and developed using luminescent solution (cat. no. PK10002; ProteinTech Group, Inc.). The protein bands were observed using a MicroChemi bioimaging system (DNR Imaging Systems Ltd.). Image J software (v1.52; National Institutes of Health) was used to measure the grey value and quantify the results.

### 2.7 RNA extraction and real‐time polymerase chain reaction (PCR)

Processed cells were placed on ice and washed with PBS three times. Total RNA was extracted using Trizol reagent according to the manufacturer's instructions. The mRNA was reverse transcribed using the PrimeScript RT Reagent Kit with gDNA Eraser (RR047A; Takara Biotechnology Co., Ltd.). The miRNA was reverse transcribed using the Mir‐X miRNA qRT‐PCR TB Green® Kit (638,314; Clontech). Real‐time PCR was performed using an SYBR Green Kit and a Roche Light Cycler® 480 II system (Roche Diagnostics). The relative expression of mRNA was analysed using *GAPDH* as an internal reference. The relative expression of target miRNA was analysed using U6 as an internal reference. Gene expression was calculated using the 2^−ΔΔCT^ method.

### 2.8 Enzyme‐linked immunosorbent assay

Post‐treatment, the cell supernatant was stored and centrifuged to remove cell debris. Secreted RANKL in the supernatant was detected using ELISA kits (R&D Systems, Inc.) according to the manufacturer's instructions. The absorbance was measured at 450 nm using an ELISA microplate reader. The concentration of secreted RANKL was determined using a standard curve.

### 2.9 Enrichment analysis and transcription factor‐promoter prediction

The MiRNA Enrichment Analysis and Annotation database (https://ccb‐compute2.cs.uni‐saarland.de/mieaa2/user_input/) was used to analyse the relevant biological processes and pathways of miRNAs which could all take Rev‐Erbα as target genes. Potential transcription factors that could target RANKL were predicted by using the databases UCSC (http://genome.ucsc.edu/) and JASPAR (https://jaspar.genereg.net/) and organizing and consolidating these data.

### 2.10 Statistical analysis

SPSS software (IBM Corp.) and PRISMA software (GraphPad Software, Inc.) were used for data analysis. Results are expressed as the mean ± standard deviation of three independent experiments. Comparison between two groups was made by *t*‐test, and comparison between multiple groups was conducted by post‐analysis of variance (anova). Results with *p* < 0.05 were considered statistically significant.

## 3 RESULTS

### 3.1 Melatonin inhibited high glucose‐induced osteoclastogenesis of RAW264.7 cells

**FIGURE 1:**
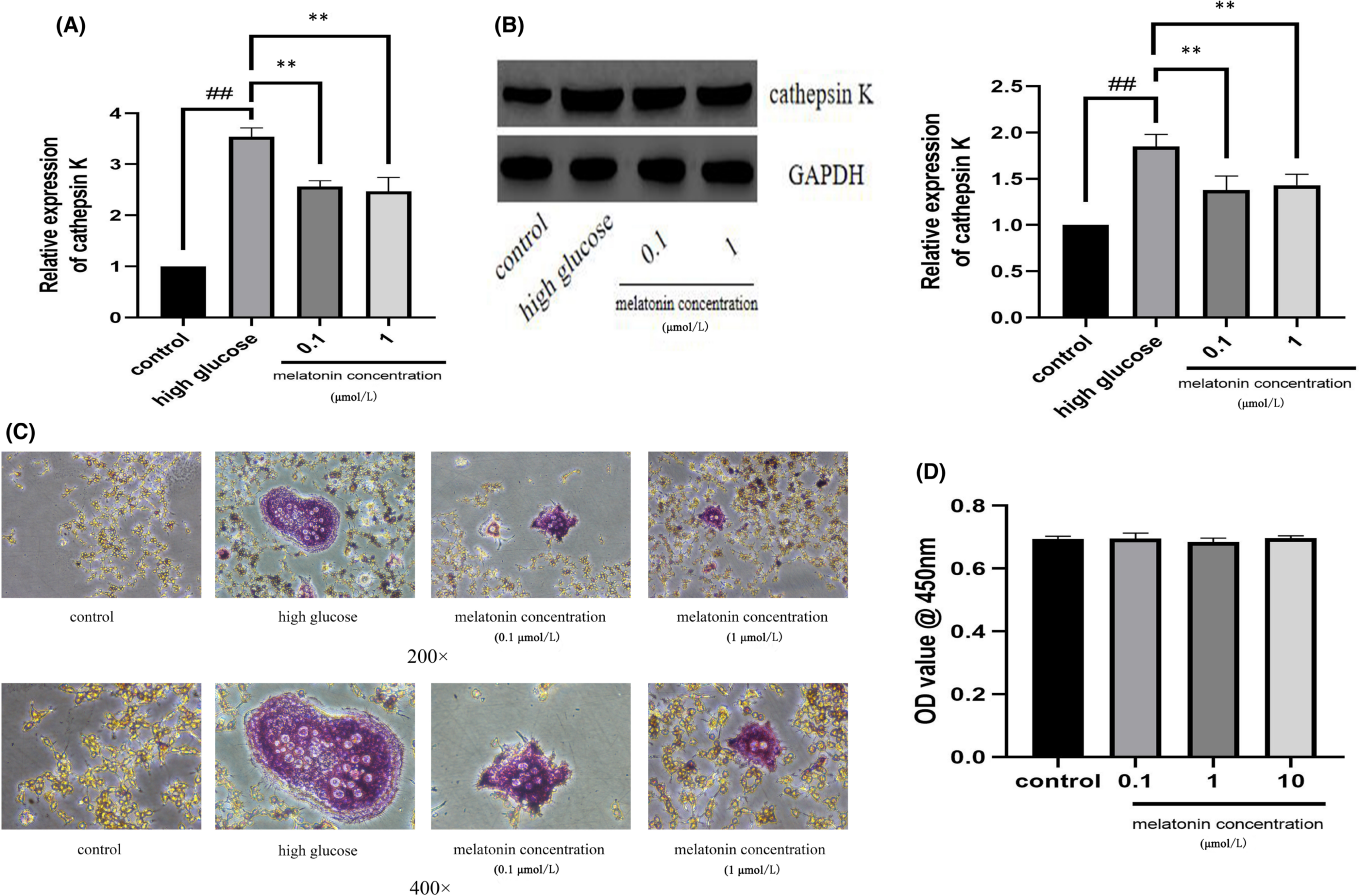
In the co‐culture system, melatonin inhibits high glucose‐induced osteoclastogenesis of Raw264.7 cells. Cathepsin K (A) mRNA and (B) protein expression in Raw264.7 cells cultured for 7 days with RANKL (30 ng/mL) and M‐CSF (10 ng/mL) with high glucose in the presence of varying concentrations (0.1 or 1 μmol/L) of melatonin. (C) Tartrate‐resistant acid phosphatase activity in Raw264.7 cells cultured for 7 days with RANKL (30 ng/mL) and M‐CSF (10 ng/mL) with high glucose in the presence of varying concentrations (0.1 or 1 μmol/L) of melatonin. Magnification, ×200 (upper panels) and ×400 (bottom panels). (D) Viability of MC3T3‐E1 cells in varying concentrations (0.1, 1, or 10 μmol/L) of melatonin. Cell viability was expressed as a percentage of the control. Data are represented as the mean ± SD (*n* = 3). **p < 0.01. OD, optical density; RANKL, receptor activator of nuclear factor κ B ligand; M‐CSF, macrophage colony‐stimulating factor

To study the effect of melatonin on osteoclastogenesis using the co‐culture system, we evaluated the mRNA and protein expression of cathepsin K^45^, ^46^ (Figure 1A,B). The expression of cathepsin K at both the mRNA and protein levels increased in RAW264.7 cells treated with high glucose (25 mmol/L)^35^, ^36^; however, these decreased after treatment with 0.1 and 1 μM melatonin. We then studied osteoclastogenesis using TRAP staining 48 h after melatonin treatment (Figure 1C). At high glucose concentrations, more TRAP‐positive cells were observed in the osteoblast–osteoclast co‐culture system; however, the number decreased after melatonin treatment. We also assessed the cytotoxicity of melatonin using a CCK‐8 assay (Figure 1D). The results showed that melatonin was not cytotoxic to MC3T3‐E1 or RAW264.7 cells, even after 48 h of treatment. However, melatonin significantly inhibited high glucose‐induced osteoclastogenesis of RAW264.7 cells in the co‐culture system.

### 3.2 Melatonin inhibited RANKL/OPG expression and secretion in osteoblasts

**FIGURE 2:**
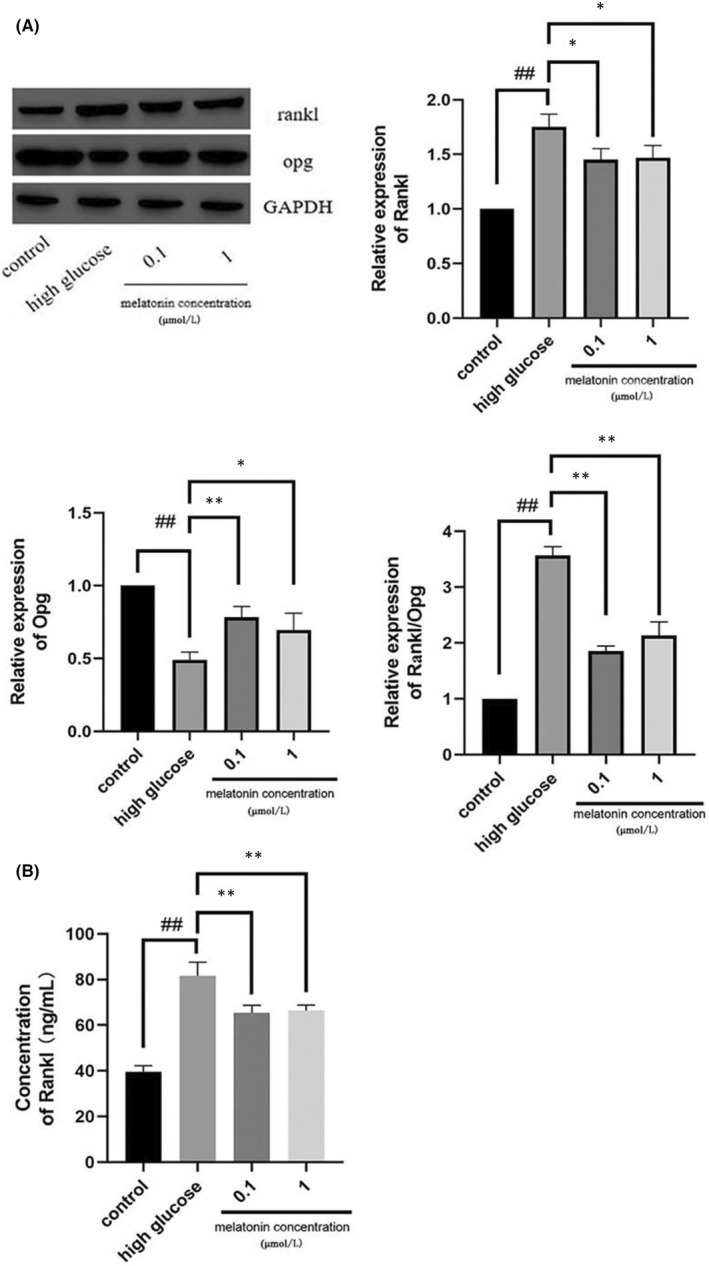
In the co‐culture system, melatonin upregulates the expression of OPG and decreases the expression and secretion of RANKL. The expression (A) and secretion (B) of RANKL and OPG in MC3T3‐E1 cells cultured for 7 days with RANKL (30 ng/mL) and M‐CSF (10 ng/mL) with high glucose in the presence of varying concentrations (0.1 or 1 μmol/L) of melatonin. Data are represented as the mean ± SD (*n* = 3). **p < 0.01. OPG, Osteoprotegerin; RANKL, receptor activator of nuclear factor κ B ligand; M‐CSF, macrophage colony‐stimulating factor

RANKL and OPG expressed and secreted by osteoblasts are closely associated with osteoclastic differentiation. The status of RANKL and OPG in osteoblasts and the cell supernatant of the co‐culture system were analysed post‐high‐glucose treatment by performing western blotting and ELISA, respectively. We determined that high glucose (25 mmol/L) promoted the expression and secretion of RANKL but inhibited the expression of OPG (Figure 2A,B). After 0.1 and 1 μM melatonin treatment for 48 h, the expression and secretion of RANKL decreased, while expression of OPG increased (Figure 2A,B).

### 3.3 Melatonin increased Rev‐Erbα expression in MC3T3‐E1 cells

**FIGURE 3:**
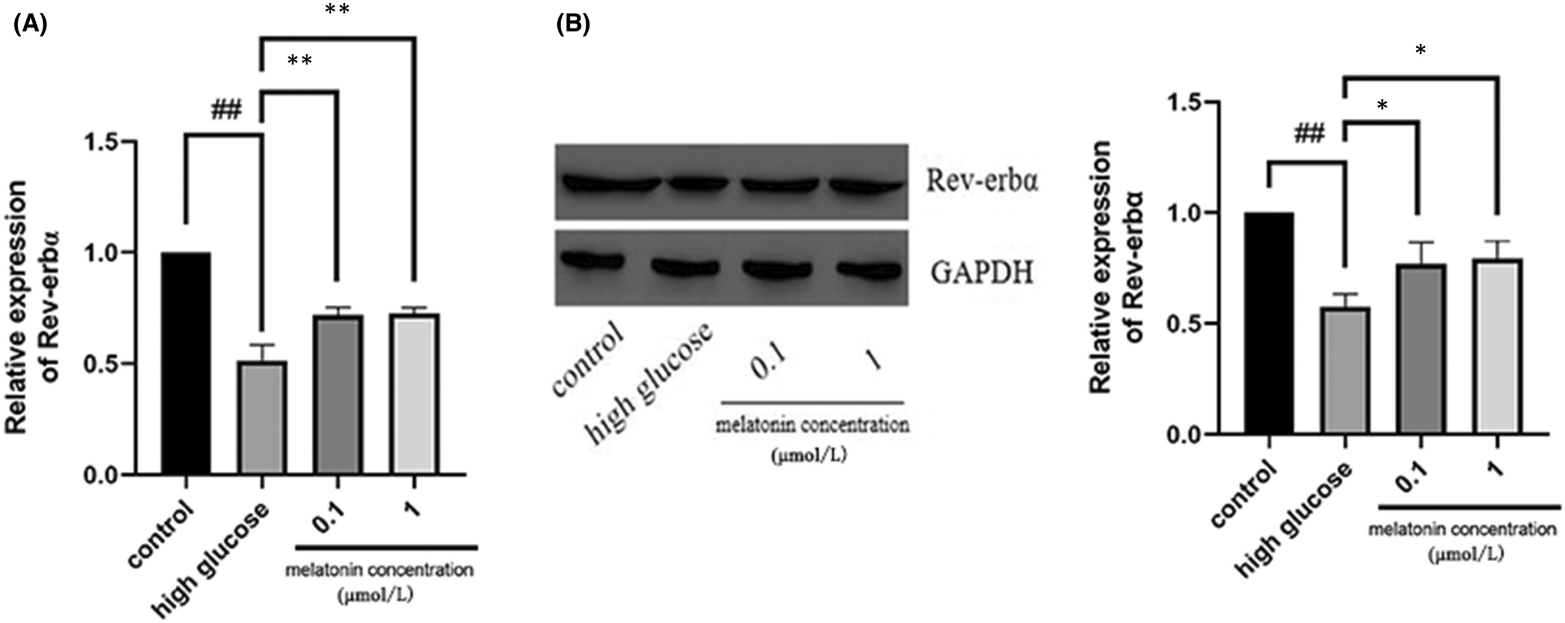
Rev‐erbα expression in MC3T3‐E1 cells is augmented by melatonin. Rev‐erbα (A) mRNA and (B) protein expression in MC3T3‐E1 cells cultured for 7 days with RANKL (30 ng/mL) and M‐CSF (10 ng/mL) with high glucose in the presence of varying concentrations (0.1 or 1 μmol/L) of melatonin for 48 h. Data are represented as the mean ± SD (*n* = 3). **p < 0.01. RANKL, receptor activator of nuclear factor κ B ligand; M‐CSF, macrophage colony‐stimulating factor; Rev‐erbα/NR1D1, nuclear receptor subfamily 1 group D member 1

During high glucose‐induced osteoclast differentiation of RAW264.7 cells, mRNA, and protein expression levels of Rev‐Erbα in MC3T3‐E1 cells were inhibited. However, 0.1 and 1 μM melatonin treatment significantly increased Rev‐Erbα expression at both the mRNA and protein levels (Figure 3A,B).

### 3.4 Rev‐Erbα activation enhanced the inhibitory effect of melatonin on osteoclastogenesis

**FIGURE 4:**
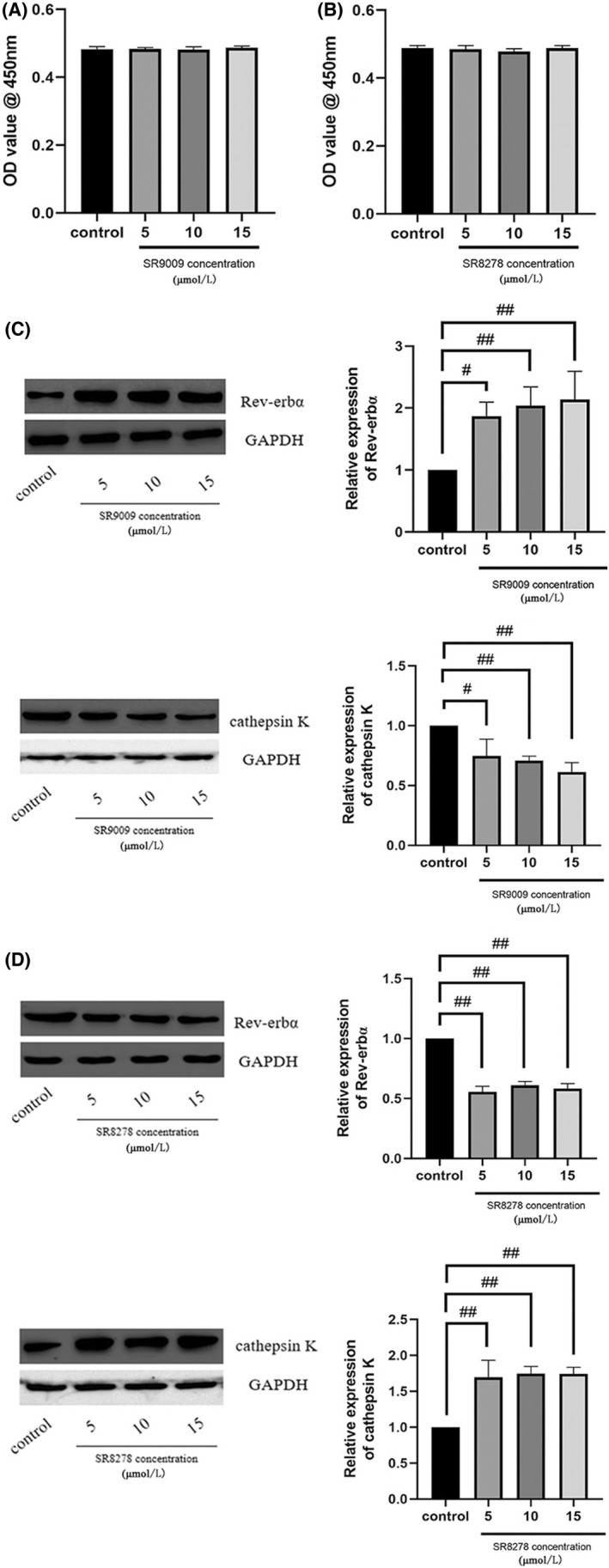
Rev‐erbα activation increases the inhibitory effect of melatonin on Raw264.7 cell osteoclastogenesis, whereas inhibition of Rev‐erbα produces the opposite effect. Viability of MC3T3‐E1 cells in the presence of varying concentrations of (A) SR9009 and (B) SR8278 (5, 10, and 15 μmol/L). Cell viability was expressed as a percentage of the control. Rev‐erbα expression in MC3T3‐E1 cells and cathepsin K expression in Raw264.7 cells cultured for 7 days with RANKL (30 ng/mL) and M‐CSF (10 ng/mL) with high glucose, and for 48 h with 1 μmol/L melatonin in the presence of varying concentrations of (C) SR9009 and (D) SR8278 (5, 10, and 15 μmol/L). Data are represented as the mean ± SD (*n* = 3). ^#^ *p* < 0.05 and ^##^ *p* < 0.01. OD, optical density; RANKL, receptor activator of nuclear factor κ B ligand; M‐CSF, macrophage colony‐stimulating factor; Rev‐erbα/NR1D1, nuclear receptor subfamily 1 group D member 1

The Rev‐Erbα agonist sr9009 and antagonist sr8278 were used in the co‐culture system to evaluate the effect of melatonin on MC3T3‐E1 cells and on osteoclast differentiation of RAW264.7 cells after high glucose treatment. Consistent with previous studies, we found that neither sr9009 nor sr8278 had cytotoxic effects on MC3T3‐E1 cells after treatment for 48 h, as evaluated using the CCK‐8 assay (Figure 4A,B). Western blotting results showed that melatonin inhibited osteoclastogenesis more significantly in MC3T3‐E1 cells treated with Rev‐Erbα agonist sr9009 than without sr9009 (Figure 4C), while Rev‐Erbα antagonist sr8278 produced the opposite effect (Figure 4D).

### 3.5 Rev‐Erbα activation suppressed RANKL/OPG expression and secretion

**FIGURE 5:**
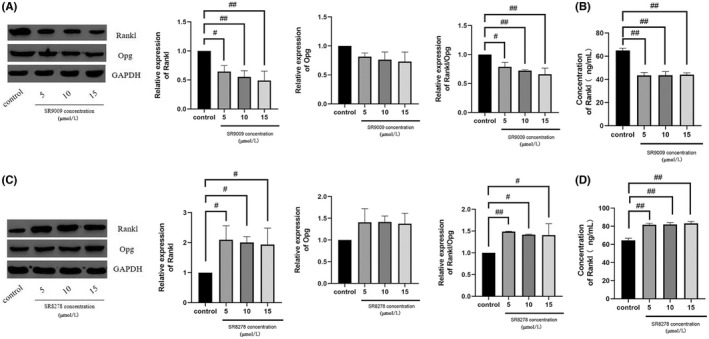
Rev‐erbα activation decreases the expression and secretion of RANKL/OPG. The expression and secretion of OPG and RANKL in MC3T3‐E1 cells cultured for 7 days with RANKL (30 ng/mL) and M‐CSF (10 ng/mL) with high glucose, and for 48 h with 1 μmol/L melatonin in the presence of varying concentrations of (A, B) SR9009 and (C, D) SR8278 (5, 10, and 15 μmol/L). Data are represented as the mean ± SD (*n* = 3). ^#^ *p* < 0.05 and ^##^ *p* < 0.01. OPG, Osteoprotegerin; RANKL, receptor activator of nuclear factor κB ligand; M‐CSF, macrophage colony‐stimulating factor; Rev‐erbα/NR1D1, nuclear receptor subfamily 1 group D member 1

The Rev‐Erbα agonist sr9009 and antagonist sr8278 were also used in the co‐culture system to evaluate the effect of Rev‐Erbα on the expression and secretion of RANKL and OPG in high glucose‐treated MC3T3‐E1 cells. Western blotting and ELISA results revealed that melatonin significantly suppressed RANKL and OPG expression and secretion from MC3T3‐E1 cells treated with sr9009 compared to that of control cells (Figure 5A,B), while sr8278 induced the opposite effect (Figure 5C,D).

### 3.6 Mir‐882 exerted its effects through Rev‐Erbα

**FIGURE 6:**
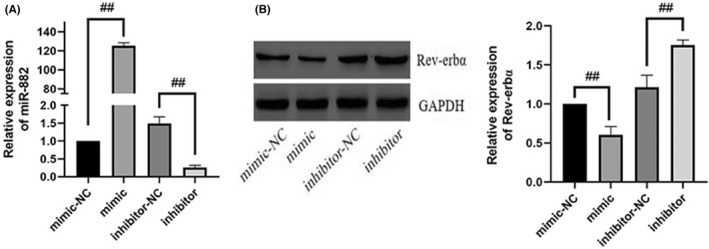
mir‐882 targets Rev‐erbα. (A) Transfection efficiency of mir‐882 mimics and inhibitors. (B) Rev‐erbα protein expression after transfection with mir‐882 mimics, inhibitors, and corresponding NCs. Data are represented as the mean ± SD (*n* = 3). *p < 0.05 and **p < 0.01. miRNA/mir, microRNA; NC, negative control; Rev‐erbα/NR1D1, nuclear receptor subfamily 1 group D member 1

Rev‐Erbα, as a target gene of mir‐882, has been shown in previous studies to mediate some of the biological effects of mir‐882, such as inhibiting osteoclastogenesis. Accordingly, we transfected MC3T3‐E1 cells with mir‐882 mimics, inhibitors, or NC (Figure 6A), co‐cultured these with RAW264.7 cells, and then evaluated the expression of mir‐882 and Rev‐Erbα. The results indicated the level of mir‐882 expression in MC3T3‐E1 cells was significantly higher in mir‐882 mimic‐treated cells than that in NC cells. In contrast, the expression of mir‐882 was lower in mir‐882 inhibitor‐treated MC3T3‐E1 cells than that of NC cells. We examined the expression of Rev‐Erbα to determine whether it was regulated by mir‐882 (Figure 6B). Transfection of MC3T3‐E1 cells with mir‐882 mimics significantly reduced the expression level of Rev‐Erbα protein in MC3T3‐E1 cells, while inhibition of mir‐882 had the opposite effect.

### 3.7 Melatonin downregulated mir‐882 expression and mir‐882 inhibition suppressed osteoclastogenesis

**FIGURE 7:**
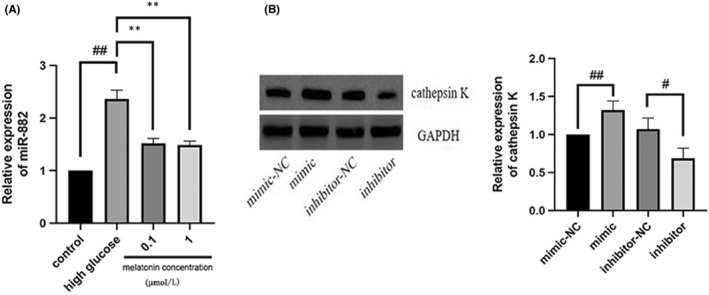
Melatonin downregulates mir‐882 expression in high glucose‐treated MC3T3‐E1 cells, while overexpression of mir‐882 in MC3T3‐E1 cells promotes osteoclastogenesis of Raw264.7 cells. (A) mir‐882 expression in MC3T3‐E1 cells cultured for 7 days with RANKL (30 ng/mL) and M‐CSF (10 ng/mL) with high glucose in the presence of varying concentrations (0.1 or 1 μmol/L) of melatonin. (B) Cathepsin K expression in Raw264.7 cells after MC3T3‐E1 cell transfection with mir‐882 mimics, inhibitors, and corresponding NCs. Data are represented as the mean ± SD (*n* = 3). *p < 0.05 and **p < 0.01. RANKL, receptor activator of nuclear factor κ B ligand; M‐CSF, macrophage colony‐stimulating factor; mir, microRNA; NC, negative control

Since mir‐882 targeted Rev‐Erbα expression in MC3T3‐E1 cells, we further explored its influence on osteoclastogenesis. To elucidate the role of mir‐882 in melatonin‐mediated inhibition of high glucose‐induced osteoclastogenesis, we evaluated the expression of mir‐882 in MC3T3‐E1 cells using real‐time PCR (Figure 7A). The results indicated that melatonin reduced the expression of mir‐882 after high glucose (25 mmol/L) treatment. Next, we transfected MC3T3‐E1 cells with mir‐882 mimics, inhibitors, or NCs, co‐cultured the transfected cells with RAW264.7 cells, and then evaluated the expression of cathepsin K in the RAW264.7 cells. Compared with that of NC‐transfected cells, overexpression of mir‐882 increased the expression of cathepsin K, while transfection with mir‐882 inhibitors decreased the expression of cathepsin K (Figure 7B). This suggested that mir‐882 inhibiton may inhibit osteoclastogenesis.

### 3.8 mir‐882 inhibition suppressed RANKL/OPG expression and secretion

**FIGURE 8:**
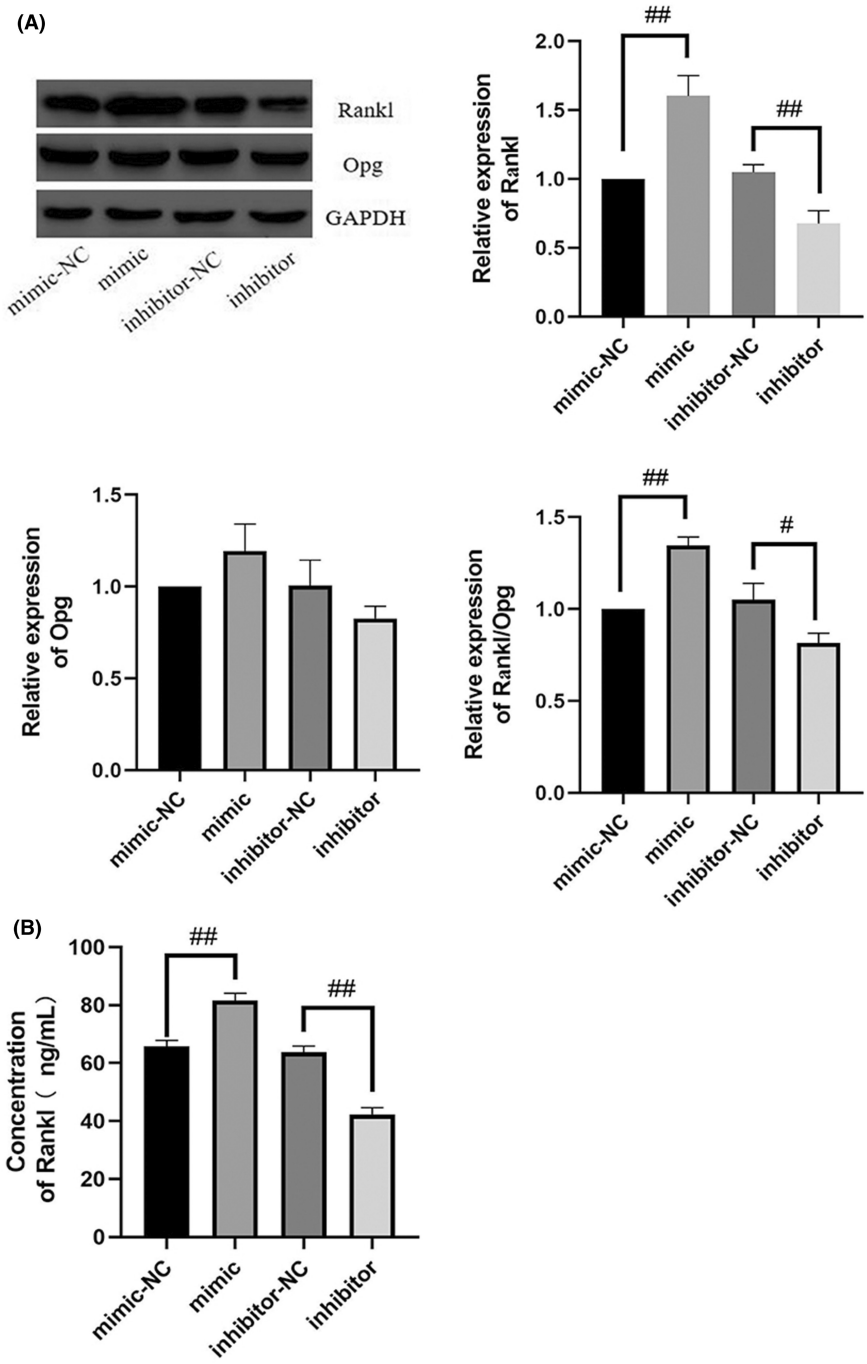
Inhibition of mir‐882 inhibits expression and secretion of RANKL/OPG. The expression (A) and secretion (B) of OPG and RANKL in MC3T3‐E1 cells after transfection with mir‐882 mimics and inhibitors and the corresponding NCs. Data are represented as the mean ± SD (*n* = 3). *p < 0.05 and **p < 0.01. OPG, Osteoprotegerin; RANKL, receptor activator of nuclear factor κ B ligand; M‐CSF, macrophage colony‐stimulating factor; mir, microRNA; NC, negative control

We further explored the capability of mir‐882 to modulate the expression and secretion of RANKL/OPG, thereby affecting osteoclastogenesis. We transfected MC3T3‐E1 cells with mir‐882 mimics, inhibitors, or NCs, co‐cultured the transfected cells with RAW264.7 cells, and after that examined the expression and secretion of RANKL/OPG from the transfected MC3T3‐E1 cells (Figure 8A,B). The results showed that transfection with mir‐882 mimics increased RANKL/OPG expression and secretion in MC3T3‐E1 cells, while mir‐882 inhibitors decreased expression and secretion. Collectively, our results showed that downregulation of mir‐882 in MC3T3‐E1 cells hindered osteoclastogenesis, while mir‐882 overexpression may promote osteoclastogenesis.

### 3.9 Rev‐Erbα regulates Rankl expression via indirect binding to the Rankl promoter

**FIGURE 9:**
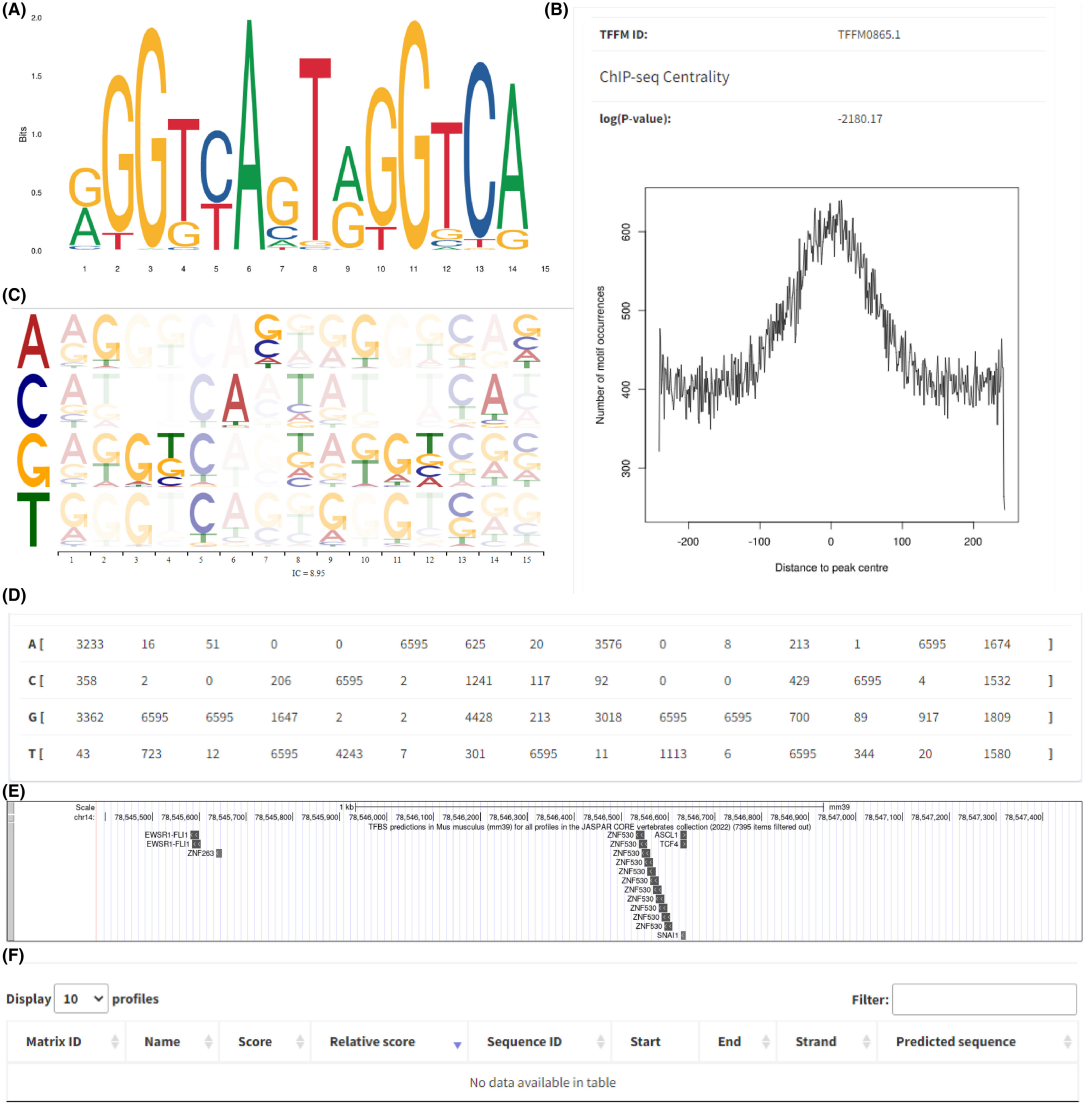
RANKL expression was regulated by Rev‐Erbα but not by direct binding to RANKL. Rev‐Erbα Sequence logo (A), frequency matrix (B), TFFM first order summary logos (C) and TFFM peak logo (D). Potential transcription factors that could target RANKL (E). No putative sites (F) were predicted in JASPAR

Rev‐Erbα reportedly regulates the expression of various target genes as a transcription factor repressor; therefore, it is likely that its inhibition of osteoclastogenesis occurs through the deactivation of downstream target gene transcription. We explored whether Rev‐Erbα regulates Rankl expression via direct binding to the Rankl promoter. First, we determined the characteristics of Rev‐Erbα using the JASPAR database, including sequence logo (Figure 9A), frequency matrix (Figure 9B), TFFM first order summary logos (Figure 9C), and TFFM peak logo (Figure 9D). After that, we used the NCBI Gene database to determine the location of the promoter of Rankl, Chr 14:78545384–78,547,483. Next, the UCSC database was used to predict potential transcription factors that could target Rankl. Unfortunately, we found that Rev‐Erbα was not included in the list of potential transcription factors of Rankl (Figure 9E). Finally, the JASPAR database was used again to analyse the sequences of Rev‐Erbα and the promoter of Rankl. Consistent with the UCSC database results, a total of 0 putative sites were predicted with a relative profile score threshold of 80% (Figure 9F).

### 3.10 Enrichment analysis

**FIGURE 10:**
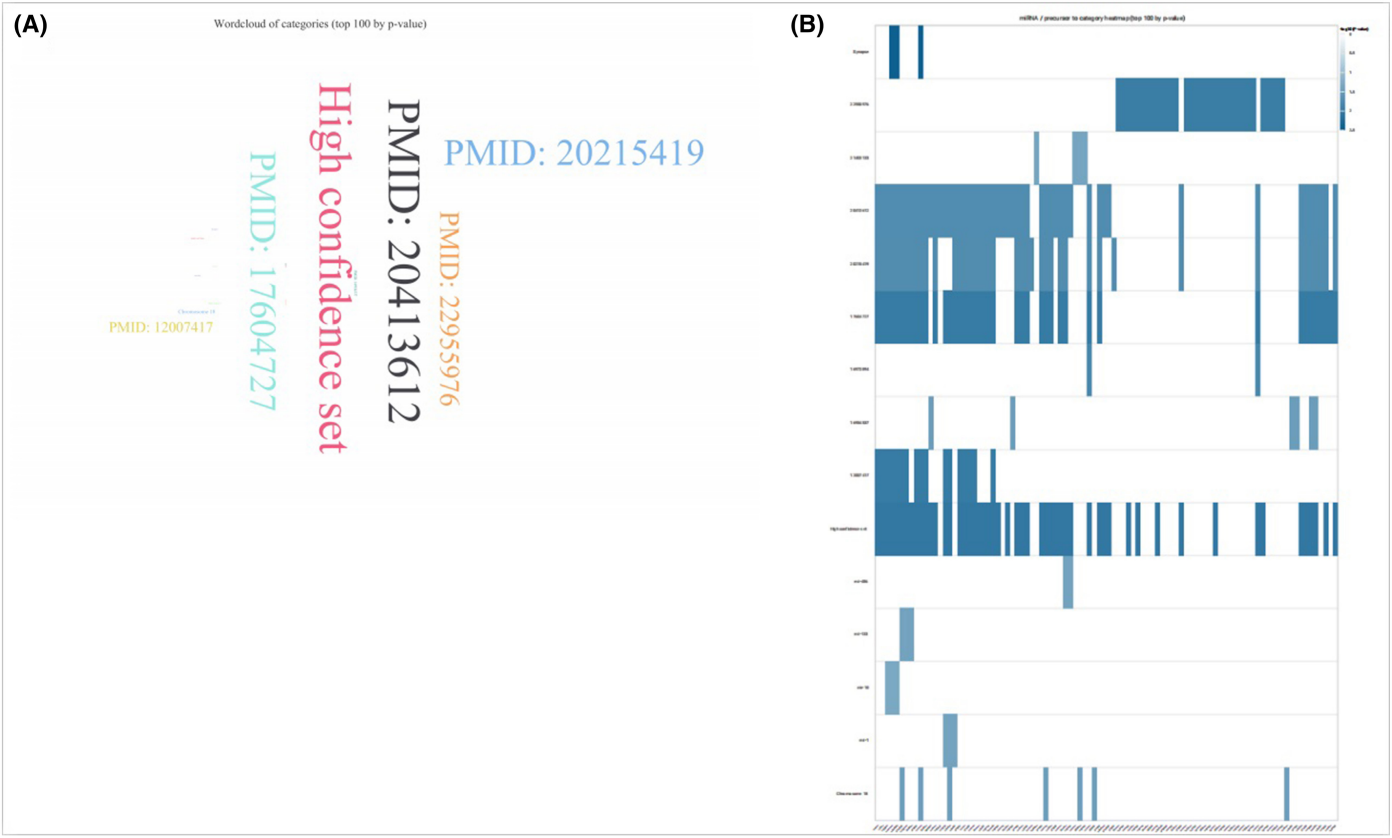
Enrichment analysis. Wordcloud of categories (top 100 by p‐value) (A). miRNA/precursor to category heatmap (B)

**FIGURE 11:**
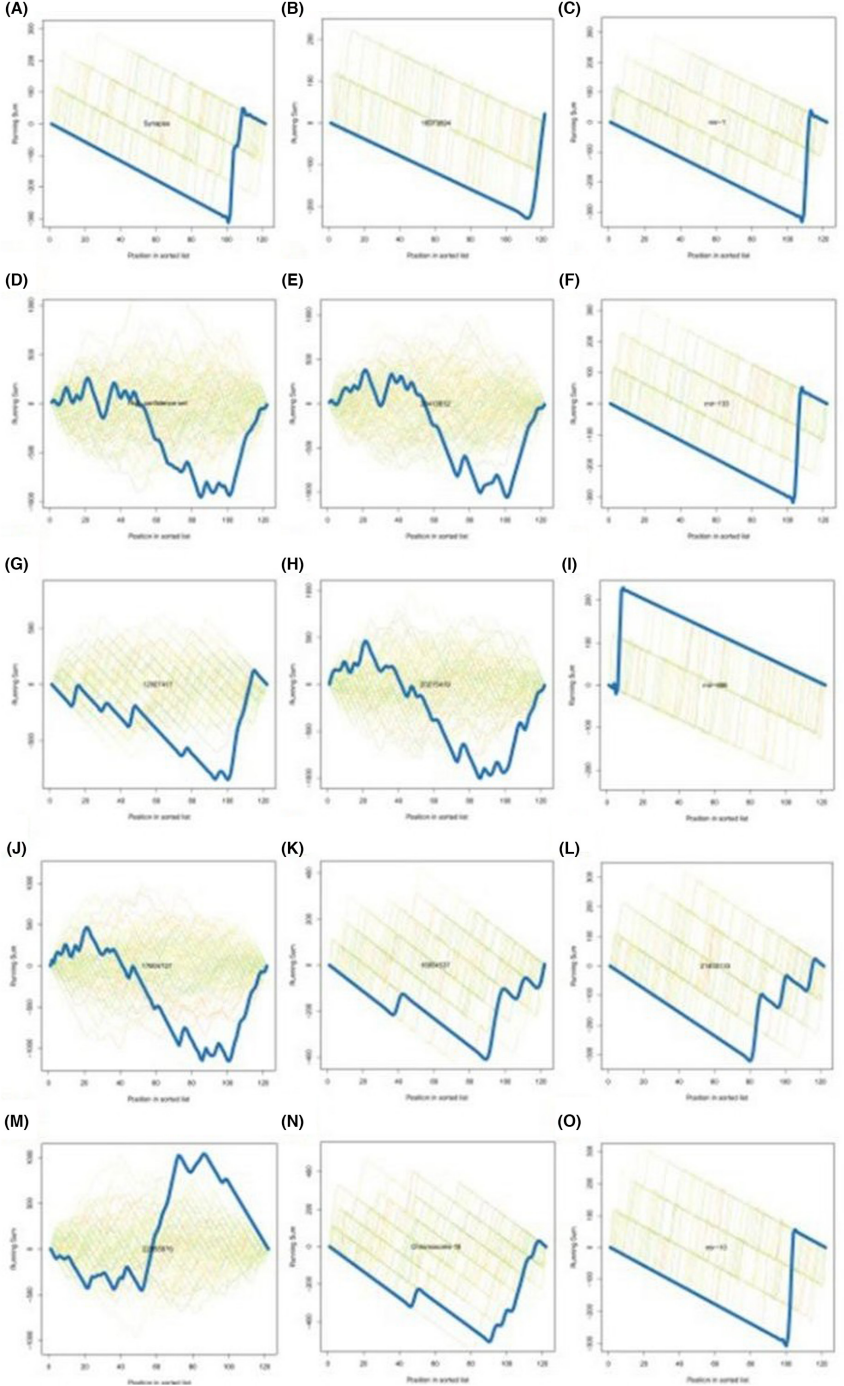
Enrichment analysis. Localization (RNALocate) Synapse (A); Confidence (miRBase) High confidence set (B); Pubmed (miRBase) 12,007,417 (C); Pubmed (miRBase) 17,604,727 (D); Pubmed (miRBase) 22,955,976 (E); Pubmed (miRBase) 16,973,894 (F); Pubmed (miRBase) 20,413,612 (G); Pubmed (miRBase) 20,215,419 (H); Pubmed (miRBase) 16,954,537 (I); Chromosomal location (miRBase) Chromosome 18 (J); Family (miRBase) mir‐1 (K); Family (miRBase) mir‐133 (L); Family (miRBase) mir‐486 (M); Pubmed (miRBase) 21,403,133 (N); Family (miRBase) mir‐10 (O)

The potential miRNAs that cooperate biological pathways always require collegial effects to bring about consolidated biological functions. Biological process and pathway analysis can make us a better understanding of miRNA function. Wordcloud of categories (top 100 by *p*‐value) was shown in Figure 10A. In Figure 10B, miRNA/precursor to category heatmap (top 100 by *p*‐value) was listed. Localization (RNALocate), Confidence (miRBase), Pubmed (miRBase), Chromosomal location (miRBase) and Family (miRBase) were exhibited from Figure 11A–O.

## 4 DISCUSSION

Diabetes can lead to osteoporosis due to the high glucose environment inhibiting osteogenesis.^47^, ^48^, ^49^ Moreover, diabetes can promote osteoclastogenesis.^50^, ^51^, ^52^ These processes are closely associated with regulating RANKL/OPG expression and secretion under high glucose concentrations. In MLO‐Y4 cells, high glucose can promote the expression and secretion of RANKL and inhibit the expression and secretion of OPG.^53^ This is consistent with the results from a rat model of diabetic osteoporosis reported by Qi et al.^54^ and those obtained from human periodontal ligament fibroblasts^55^ and vascular smooth muscle cells.^56^ However, contradictory results have also been reported from a few studies. Qu et al. reported that high glucose inhibits osteoclastogenesis in osteoclast precursor cells isolated from Sprague–Dawley rats, while high glucose and palmitate (or ester) promote osteoclastogenesis.^57^ Similarly, Chang et al. suggested that high glucose reduces RANKL/OPG expression in vascular smooth muscle cells (A7r5) and causes vascular calcification.^58^

Based on the reports that a high‐glucose environment and biological rhythm influence each other,^59^, ^60^, ^61^, ^62^ we proposed that high glucose may regulate RANKL/OPG expression by altering the biological rhythm and expression of Rev‐Erbα. The existence of circadian rhythms has been reported in bone tissue, and bone disorders are associated with the occurrence of osteoporosis.^63^, ^64^, ^65^, ^66^, ^67^ Furthermore, Rev‐Erbα, as a circadian regulatory protein, is involved in the regulation of bone metabolism. Recent studies have shown that Rev‐Erb inhibits osteogenic differentiation^68^, ^69^, ^70^ and suppresses osteoclast differentiation.^69^, ^71^ These results suggest that Rev‐Erb exhibits biological characteristics of a transcription inhibitor.^71^, ^72^, ^73^, ^74^, ^75^, ^76^, ^77^, ^78^ However, our analysis using JASPAR and the UCSC database confirmed that Rev‐Erbα regulates Rankl expression via indirect binding to the Rankl promoter, suggesting an underlying mechanism or signalling pathway between Rev‐Erb and Rankl. Since osteoblasts and osteoclasts co‐exist and interact to maintain bone homeostasis in vivo,^79^, ^80^, ^81^ the co‐culture of osteoblasts and osteoclasts is a reliable model system to simulate the in vivo environment. Thus, the results obtained in our in vitro system are expected to translate to in vivo culture conditions but should be validated in animal models or primary cell culture‐based systems in future studies.

However, in the present study, we mainly focused on MC3T3‐E1/Raw264.7 cells co‐culture system induced by high glucose in vitro and we think that in vitro experiment though not optimal, should be sufficient to draw the conclusion that melatonin inhibits osteoclastogenesis via RANKL/OPG suppression mediated by Rev‐Erbα in osteoblasts.

In this study, we focus on the fact that melatonin inhibits osteoclastogenesis via RANKL/OPG suppression mediated by Rev‐Erbα in osteoblasts. Rev‐Erbα is not only a circadian clock protein, but also a target of the circadian hormone, melatonin. We think that no time course of expression, though not optimal, should be sufficient to conclude that melatonin inhibits osteoclastogenesis via RANKL/OPG suppression mediated by Rev‐Erbα in osteoblasts.

In the co‐culture system, osteoblasts were not allowed cell‐to‐cell contact with RAW264.7 cells. Melatonin does have a direct impact on RAW264.7 cell viability or osteoclastogenesis. In the co‐culture system, there is indeed such a role that melatonin does directly impact RAW264.7 cell viability or osteoclastogenesis. However, to demonstrate the change of RANKL/OPG in osteoblasts, we studied the molecular biology of the two kinds of cells and concluded that the addition of melatonin reduced RANKL/OPG in osteoblasts. It is well known that RANKL/OPG can promote osteoclast differentiation.

To the best of our understanding, certain prior studies have already provided an explanation for melatonin receptor.^82^ Moreover, in the present study, we primarily focused on the fact that melatonin inhibits osteoclastogenesis via RANKL/OPG suppression mediated by Rev‐Erbα in osteoblasts. The obtained results revealed the conclusion, which comprehensively explains the issue at hand. The fact that no melatonin receptor related experiments have been conducted should suffice to draw conclusions, even though it is not ideal.

## 5 CONCLUSION

High glucose promoted osteoclast differentiation in our osteoblast–osteoclast co‐culture system, while melatonin partially reversed the high glucose effects. This suggested that melatonin may be beneficial in treating osteoporosis by inhibiting osteoclast differentiation. Moreover, we found that the melatonin‐induced reversal was mediated by the mir‐882/Rev‐Erbα axis, which reduced the expression and secretion of RANKL/OPG in osteoblasts, thereby inhibiting osteoclastogenesis. This study provides a new theoretical basis for diabetic osteoporosis and reveals the potential of melatonin for treating diabetic osteoporosis by inhibiting osteoclastogenesis.

## AUTHOR CONTRIBUTIONS

**Yihao Tian:** Conceptualization (lead). **Jian Ming:** Writing – review and editing (lead).

## CONFLICT OF INTEREST

The authors declare that the study was conducted in the absence of any commercial or financial relationships that could be construed as a potential conflict of interest.

## Data Availability

The data that support the findings of this study are available from the corresponding author upon reasonable request.
